# Web-Based Assessments of Physical Activity in Youth: Considerations for Design and Scale Calibration

**DOI:** 10.2196/jmir.3626

**Published:** 2014-12-01

**Authors:** Pedro F Saint-Maurice, Gregory J Welk

**Affiliations:** ^1^Iowa State UniversityDepartment of KinesiologyAmes, IAUnited States; ^2^University of MinhoSchool of PsychologyCIPsiBragaPortugal

**Keywords:** measurement, questionnaire, Youth Activity Profile

## Abstract

This paper describes the design and methods involved in calibrating a Web-based self-report instrument to estimate physical activity behavior. The limitations of self-report measures are well known, but calibration methods enable the reported information to be equated to estimates obtained from objective data. This paper summarizes design considerations for effective development and calibration of physical activity self-report measures. Each of the design considerations is put into context and followed by a practical application based on our ongoing calibration research with a promising online self-report tool called the Youth Activity Profile (YAP).

We first describe the overall concept of calibration and how this influences the selection of appropriate self-report tools for this population. We point out the advantages and disadvantages of different monitoring devices since the choice of the criterion measure and the strategies used to minimize error in the measure can dramatically improve the quality of the data. We summarize strategies to ensure quality control in data collection and discuss analytical considerations involved in group- vs individual-level inference. For cross-validation procedures, we describe the advantages of equivalence testing procedures that directly test and quantify agreement. Lastly, we introduce the unique challenges encountered when transitioning from paper to a Web-based tool. The Web offers considerable potential for broad adoption but an iterative calibration approach focused on continued refinement is needed to ensure that estimates are generalizable across individuals, regions, seasons and countries.

## Introduction

Physical activity behaviors can be assessed with a variety of techniques, but each has inherent limitations [1]. Researchers have increasingly used objective monitoring devices to capture ambulatory movement [2], but there is a fundamental need to also improve the utility of self-report instruments that can be more effectively deployed in Web-based applications in a cost effective way. The sophistication (and accuracy) of objective monitoring devices has increased dramatically in recent years through the use of new technologies [3,4] and incremental advances in calibration methodologies [5-11]. Surprisingly, relatively few efforts have been made to improve the utility of more subjective, self-report measures. The *Journal of Physical Activity & Health* published the outcomes of the Measurement of Active and Sedentary Behaviors: Closing the Gaps in Self-Report Methods meeting sponsored by the National Institutes of Health that addressed a series of best practices and challenges encountered when working with self-report tools for physical activity [12]. One of the limitations of self-report instruments is a general lack of accuracy; however, the use of robust calibration procedures and implementation of Web-based applications offer considerable promise for improving the validity and utility of self-report measures [12-20]. A specific advantage is that algorithms from these methods can be directly embedded within existing Web-based applications to streamline collection and reporting of data. For optimal effectiveness, these models must be developed, tested, and then refined through an iterative process that enables enhancements to be directly incorporated into the assessment.

Measurement error models have been used to understand error in self-reported data on food intake [21-23] and physical activity behavior [22,24,25], but this work is still in early phases. An advantage in physical activity research is that there are available criterion measures from objective monitoring devices that can be used to scale the information provided on the self-report forms. However, there are a number of challenges involved in effectively linking self-report data to objective data for calibration. There are a number of other design considerations that also influence the ability to calibrate self-report data. The present paper describes key principles and design considerations needed for effective calibration of self-reported physical activity data. This paper also addresses the potential application of online self-report tools for large-scale assessment. Examples are based on work we have done to calibrate the Youth Activity Profile (YAP), a promising online self-report measure of physical activity in youth. Although some issues are specific to this instrument, the design principles, methods of calibration, and Web applications would apply to other surveys and populations.

The YAP provides a good example for illustrating calibration design and online applications because it was developed specifically with these goals in mind. It was designed for use in school settings to make it possible to obtain accurate data from youth while also providing a valuable educational experience. The YAP uses a segmented day approach and context-related events to facilitate recall by youth. It captures the key dimensions of physical activity needed for calibration (eg, frequency, type, volume), and includes separate components to capture the context of physical activity (eg, in-school vs. out-of-school) to facilitate education and promotion.

We have established the Youth Physical Activity Measurement Study (YPAMS) to facilitate continued development and refinement of the YAP. This paper first describes the key components of the YAP because examples are based on our experience with this model. We then summarize the design features and calibration principles that are important for this type of calibration work. We conclude with a discussion of the unique considerations required for the calibration of an online version of calibrated tools in the final section.

## Development of the Youth Activity Profile

The YAP is a self-report instrument designed to capture physical activity and sedentary behavior in youth. It was based conceptually on the time-based structure of the established Physical Activity Questionnaire for Children (PAQ-C) and a related tool for adolescents (PAQ-A) [26-29]. We identified the Physical Activity Questionnaire (PAQ) as a promising platform because it was consistent with established recommendations for recall instruments (ie, uses short-term recall periods, uses contextual questions, stimulates episodic memories, and asks about overall moderate-to-vigorous physical activity, MVPA). In addition, the PAQ is a short form that can provide direct comparisons among youth of different age groups. The PAQ takes less than 15-20 minutes to be completed; therefore, this instrument is very practical. All these might explain the overall good psychometric properties of this instrument [26-28,30-33].

The YAP was designed to be a self-administered 7-day (previous week) recall questionnaire suitable for use with children in grades 4 to 12. The structure for some items maintains the conceptual idea of the PAQ, but the individual items were changed to improve calibration. Additional items were added and a whole category of sedentary time was developed. The YAP includes a total of 15 items divided into 3 sections: (1) activity at school, (2) activity outside of school, and (3) sedentary behaviors. The final version of the YAP was pilot tested and cognitive interviews (in laboratory) were performed with similarly aged participants to refine the final items. The final version of the YAP is available in Multimedia Appendix 1.

**Figure 1:**
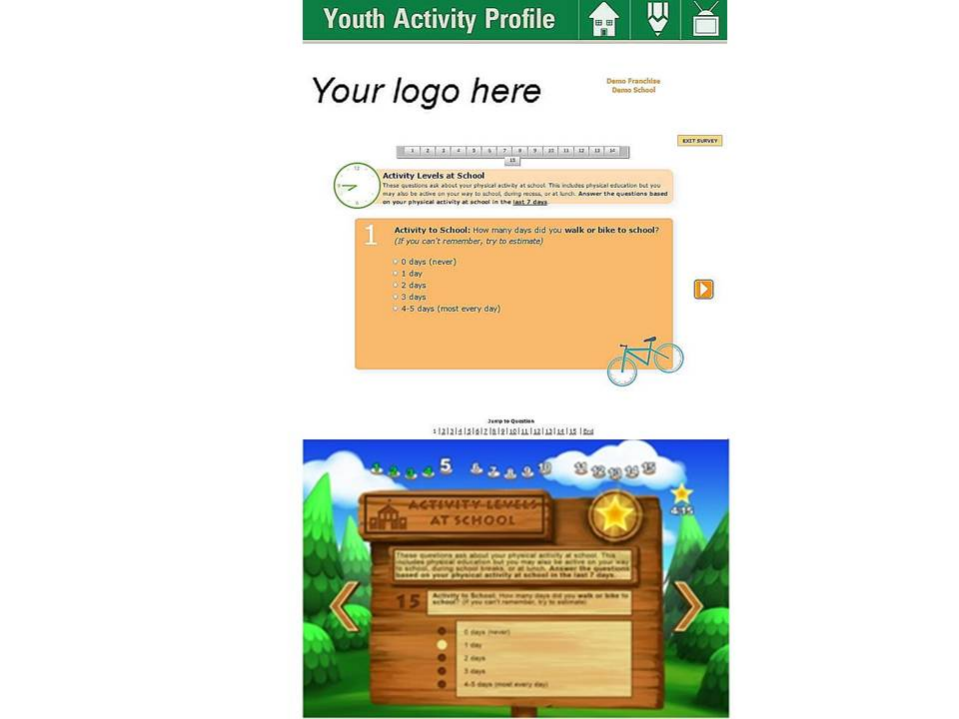
Screen capture of the online (top) and game (bottom) version of the Youth Activity Profile.

The scoring procedures used in the YAP are similar to those used in the original PAQ; however, each YAP section was developed to be scored independently (ie, items for each dimension are averaged to reflect the composite score of the respective dimension). A higher composite score for a given dimension score would reflect a higher expected activity levels/sedentary time at that same dimension. Preliminary calibration of the YAP has been conducted using a paper version (phase I and II). However, an online version of the YAP has been developed and will be used for future refinement (phase III) (Figure 1). Online versions of existent physical activity questionnaires can provide considerable advantages for both education (school) and research applications because data can be entered more easily and processed automatically to enable feedback.

## Design Considerations

### Overview

In this paper, *calibration* refers to the process of computing adjusted minutes of objectively measured physical activity from a raw self-reported physical activity index [34]. A number of factors must be considered when setting up a calibration study and each decision has ripple effects on other aspects of the design. We first describe the overall calibration design and follow with the importance of selecting an appropriate criterion measure and then describe key considerations, such as participant selection, sample size determination, measurement error, and validity of calibration algorithms for online applications. At each of these sections, we provide some contextual information of the concept/issue being discussed and then provide a practical example based on our experience with the Youth Physical Activity Measurement Study (YPAMS).

### Calibration Design

#### Background

To enhance calibration, the structure and items from self-report tools should be designed to link objective data to the subjective responses. Each question should capture a discrete period of time in which there are specific opportunities to be physically active. Descriptive studies provide documentation of settings or periods of time that capture significant amounts of physical activity [35,36]. If similar periods are identified and included in the self-report tool, it is possible to link each period to corresponding data from the activity monitor and pursue individual calibration. See sample temporal linkage provided in Table 1.

#### YPAMS Example

**Figure 2:**
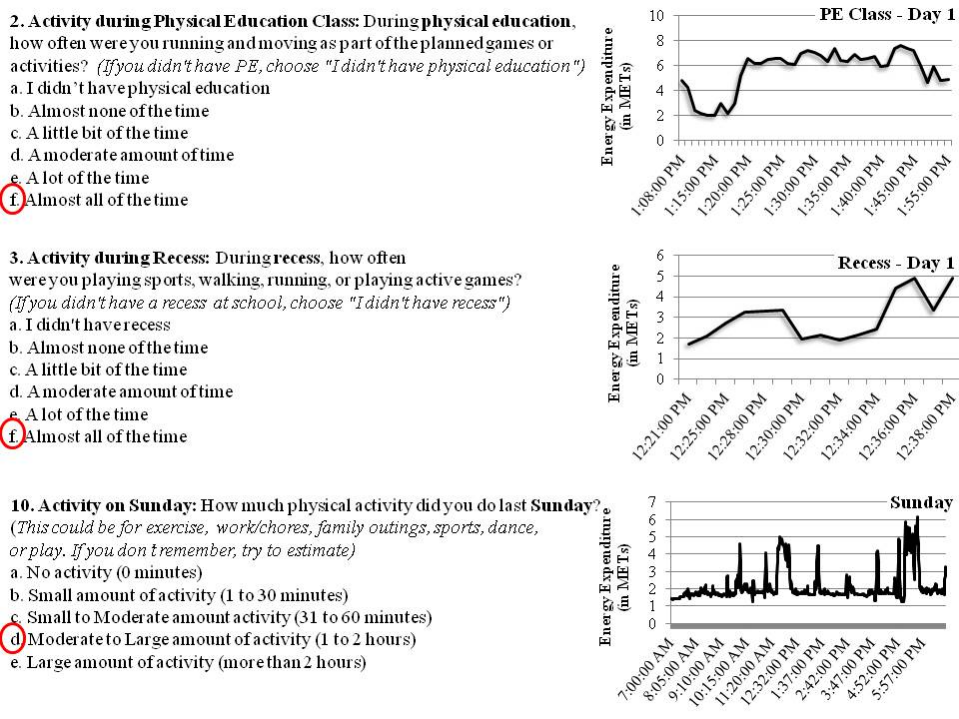
Examples of energy expenditure values measured by accelerometer for discrete time segments captured by the YAP. The data presented are from a middle school female participant enrolled in the YPAMS.

There are 5 items in the YAP that capture periods at school (ie, walk/bike to school, at recess, during physical education (PE) class, at lunch, walk/bike from school) and 5 items that capture periods at home (before school, after school, evening, Saturday and Sunday). The individual time segments are directly matched with corresponding time periods from the accelerometer (see Figure 2 for examples of student activity during PE, recess, and Sunday). The calibration process averages responses over the available number of days (and then across participants) to obtain stable estimates for each of the segments. Some time segments are standardized across days (eg, Before school, After School etc…); however, other segments such as PE, recess and lunch can vary across days so this flexibility was built into the coding.

**Table 1:** Weekly schedule used to process segmented accelerometer data.

Window	Date	Individualized time	Start time^a^	End time^a^
Before school	Every day	Yes	60 min before start time for trans to school	Start time for trans to school
Transportation to school	Every day	Yes	30 min before start time for school	Start time for school
Recess	Provided	Yes		
Physical Education	Provided	Yes		
Lunch	Every day	Yes		
Transportation from school	Every day	Yes	End time for school	30 min after end time for school
After school	Every day	Yes	End time for trans from school	6:00 pm
Evening	Every day	No	6:00 pm	10:00 pm
Saturday	Saturday	No	7:00 am	10:00 pm
Sunday	Sunday	No	7:00 am	10:00 pm

^a^ “Start” and “end” school time was obtained from schools (eg, 8:15 am-3:30 pm).

### Selection of the Criterion Measure

#### Background

There are many options available for capturing objective information about physical activity, but each has inherent advantages and disadvantages. The most widely used accelerometry-based activity monitor is the Actigraph, but a limitation of this device is that it is not possible to directly detect if a participant is wearing the device. Numerous studies have been conducted with the Actigraph, but there is still a need to develop better algorithms to capture EE [37,38] or to detect wear time [39,40].

#### YPAMS Example

For our YAP calibration work, we selected the SenseWear Armband (BodyMedia, Inc, Pittsburgh, PA, USA) as our criterion measure of physical activity. We conducted numerous studies with various accelerometry-based monitors and found the SenseWear Armband offered a number of advantages for use in calibration studies. The SenseWear Armband is a wireless pattern-recognition device that integrates motion sensor data with a variety of heat-related sensors and demographic variables to estimate the EE [41]. The multisensor nature of the monitor provides advantages over traditional accelerometry-based monitors. The heat-related sensors, for example, provide a better indicator of work (and EE) for nonlocomotor tasks and activities of daily living [42]. An additional advantage of the SenseWear Armband for field-based research is that it automatically detects periods of time in which the accelerometer is not worn (ie, removed by the participants). This is still a common source of error in physical activity studies that use more standard measures of physical activity [39].

### Characteristics of the Study Population

#### Background

The nature of the study population should be taken into account when developing recruitment and retention strategies. It is important to ensure that the sample population is representative of the larger population and that there is sufficient variability within the sample. Variability in levels of physical activity is especially important in calibration design to ensure that it can predict both low and high levels of physical activity. The calibration of a scale is also enhanced if the observed exposure values (MVPA from the SenseWear Armband) are normally distributed within each level of the covariates in the model being considered (eg, age and gender). Although controlling for covariates is necessary, one would also need to maximize the variability in your exposure variable. This task can be challenging because calibration of self-report tools are designed to capture real life physical activity patterns as opposed to common calibration activity monitors studies that are conducted in controlled environments and, therefore, allow activities to be manipulated [43,44]. One possible strategy to artificially manipulate individual activity patterns while controlling for age and gender is to recruit individuals with different activity levels.

#### YPAMS Example

**Figure 3:**
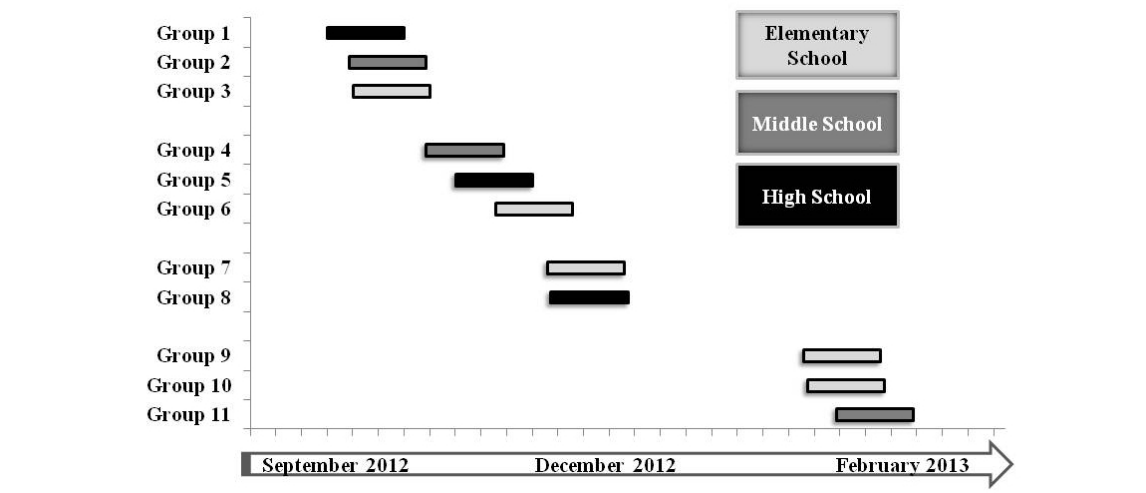
Gant chart illustrating the timeline and design for data collection of the preliminary work. Each rectangle represents a classroom grade full protocol timeline. Thick bars on the horizontal axis represent 1 calendar week. Each collection took approximately 3 weeks.

In our calibration work, we enhanced variability in activity levels by recruiting a diverse sample and by collecting activity data across seasons (eg, winter and summer). Season has been shown to influence physical activity patterns in youth [45], so the advantage of this approach is that the calibrations would capture this inherent variability and be more generalizable. The YPAMS design also counterbalanced the dates of data collection by age group (elementary, middle, and high school grades). For example, at each season, data are collected in elementary, middle, and high school participant groups composed by an approximately similar number of boys and girls, and in a counterbalanced order. Figure 3 provides an illustration of the data collection map for a full year of the YPAMS.

### Sample Size and Recruitment Issues

#### Background

Sample size calculations for physical activity calibration studies should account for important predictors of physical activity in youth. Many correlates of physical activity have been identified, but age and gender are perhaps the most important factors to consider in calibration studies. These 2 factors are known to be related with objectively measured physical activity and, therefore, will affect statistical power of the questionnaire being calibrated. Sample size estimations for calibration can be computed using multiple regression with random factors and by including 3 predictors for the full model (age, gender, and questionnaire score) at a specific alpha and power. The expected variance accounted for with the full model would depend on the nature of the assessment, but this can be determined by conducting a systematic review of the literature. In addition to statistical power, researchers should also consider typical compliance rates with activity monitoring studies and account for noncompliance by overrecruiting participants.

#### YPAMS Example

In our preliminary work of the YPAMS, we used population level variances of 0.35 for a full model with the 3 predictors described previously and 0.25 for the reduced model. These variances were defined after reviewing the literature on the agreement between self-reports and accelerometry in youth. We had compliance rates of approximately 89% and, on average, participants (including compliant and noncompliant) wore the accelerometer during 71% of awake time (ie, between 7:00 am and 10:00 pm). We learned that younger children are more willing to participate in these studies and that they tend to be more compliant compared with their older peers. We have incorporated these lessons in our YPAMS study to promote adherence and compliance with our protocol. Direct collaboration with school districts and PE teachers has facilitated recruitment and retention in our school-based testing.

### Measurement Error in the Criterion (Objective) Measure

#### Background

In the context of linear regression, measurement error can lead to a variety of flawed outcomes that can go from the attenuation of the relations between the exposure variable and health-related outcomes [46] to reversed effects when error-free measures are used [47]. The same concern applies to physical activity and, in particular, to calibration studies that have to rely on non-error-free gold-standard measures [48]. Measures obtained from activity monitor tools can lead to both random and systematic error and include (1) malfunction and handling of activity monitors, (2) day-to-day variability in activity patterns, and (3) periods of nonwear time. These sources of error can be attenuated with standardization of procedures and by scheduling periodic calibration checks of the monitors being used. Another key to reducing error is to collect data across multiple days and to define strict criteria to evaluate compliance in monitoring. Commonly used data reduction procedures include requiring valid activity counts for a minimum period of time (eg, 10 hours per day and/or 3 days per week). However, researchers can minimize the loss of data by having participants record descriptive information about nonwear bouts (eg, sports events). Data can be imputed using EE values (ie, METs) available for this purpose [49-51]. The standard MET values used for imputing missing periods were not developed to account for individual differences in the activities being considered, but the inclusion of these values avoids the alternative problem of deleting cases / time periods in which participants were not able to wear the monitors and can capture important activity that otherwise would be dismissed [52,53]. Overall, the choice of data reduction procedures will have an impact on the criterion measure outcome [54,55].

#### YPAMS Example

**Figure 4:**
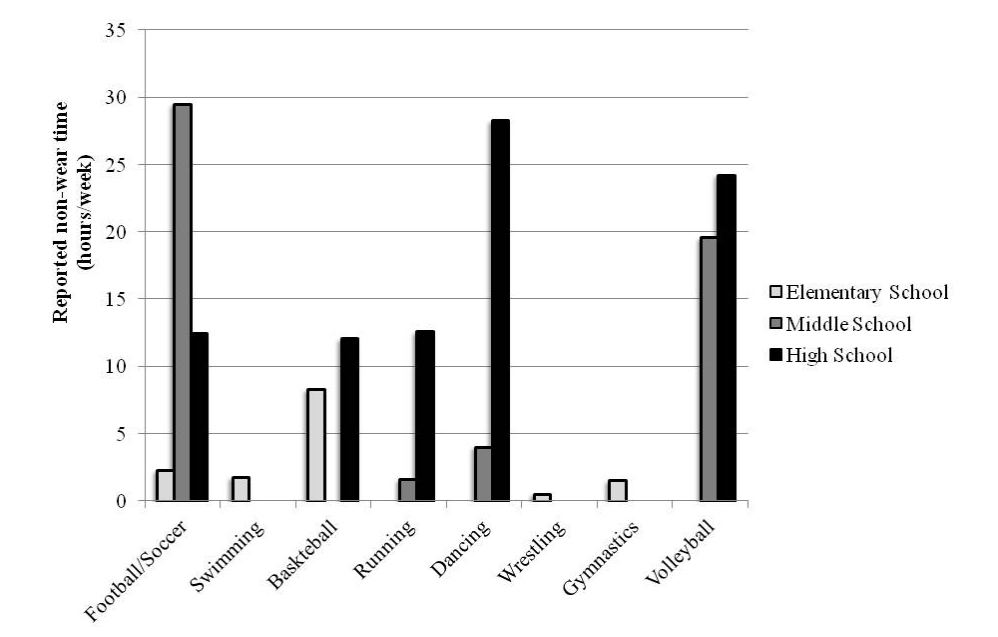
Reported nonwear time across 7 days obtained from individual logs. Data presented are from a subset of participants enrolled in the YPAMS. Results are based on a sample of 87 elementary, 27 middle school, and 29 high school students that used a SenseWear Armband for 7 consecutive days. There were 2 participants without accelerometer data who provided records of nonwear time.

In our work, we have minimized noncompliance by sending information home to parents and by calling participants early in the week to remind them about the importance of wearing the monitor. Participants were also required to have valid EE values during at least 70% of the time and at least 3 valid periods for each time window of interest (exception was made for PE class, Saturday, and Sunday segments in which participants are only required to have at least 1 valid segment). Information about nonwear time was obtained from a daily activity log and these data were used to impute standardized MET values from the compendium of physical activities [49-51]. Figure 4 provides an illustration of nonreported activity in the YPAMS study.

By imputing missing time periods (due to documented nonwear times), we have been able to maximize the available sample. We noted that nonwear time was more prevalent in older students, possibly reflecting the changes in daily physical activity patterns as children get older.

### Measurement Error in the Calibrated (Subjective) Measure

#### Background

It is important to consider sources of error in the self-report measure so that they can be quantified and modeled appropriately. Self-report tools are known to be susceptible to different sources of error, but the majority of the error can be attributed to the nature of the instrument and the individual’s subjectivity when interpreting and completing the questionnaire. For effective calibration, it is important to try to minimize error from these (and other) sources. It is important to ensure that the questions are appropriately worded and easy to understand. A formalized approach using cognitive interviews can help to identify issues with the survey before it is finalized. Error derived from the individual subjectivity can be minimized by adding recall probes and conducting quality checks after the questionnaire is completed. These 2 factors are particularly important in younger populations. Efforts to collect higher quality data from the survey will reduce error and improve calibration accuracy.

#### YPAMS Example

In our calibration work, we performed cognitive interviews by having individual face-to-face interviews with participants and asking them to think aloud as they were completing the YAP. Additionally, during our field data collection, a trained research member verified each child’s questionnaire once the questionnaire was completed. Children were asked to provide more specific information (eg, exact location, exact day, or time of the day) about the behavior reported. In some cases, this process helped verify that participants were using appropriate memories to complete the YAP (eg, recalling the previous week and not general routines).

### Individual vs Group-Level Inference

#### Background

The sources of error described previously can have different implications on physical activity output obtained from self-report tools. Most likely, this output will be used to predict individual and/or group-level physical activity. The concept of estimation at the individual and at the group level can be confusing. It is tempting to think of a group as composed of individuals and, thus, to base inference for the group on individual-level inference. However, these indicators must be examined separately. Reliable estimation of usual physical activity at the individual level is more challenging and may require multiple assessments, but individual data can be used to obtain reliable and valid estimates of the distribution of usual physical activity in the group. Several studies have demonstrated how there is substantial variability in the agreement between individual estimates of self-report data and objective measures of physical activity [56-58]. Activity collected in a clinical setting (ie, for 1 individual alone) is a good example of the application of this level of measurement. On the other hand, if physical activity is collected in a group of individuals, the error is diluted across individuals and group-level physical activity scores can actually become close to “true” physical activity scores estimated for the group. In other words, the calibration design as described in this paper can minimize the distance of the individual estimates to a line of best fit that represents the group (ie, line of best fit using least squares estimation). If standard regression assumptions hold, this procedure is known to create evenly distributed residuals that average out to a value of zero. Although individual error relies on the absolute distance between each individual observation and the line of best fit, the group estimate partials out the error to produce a close estimate of aggregate scores obtained from individuals.

#### YPAMS Example

In our development work with the YAP, we determined that individual error obtained from a calibration equation to predict minutes of MVPA from the PAQ can range from -56% to 69% of mean accelerometer values [58]. This wide range of agreement indicated that the PAQ tools might not be appropriate when used to quantify an individual’s activity. However, we also found that group-level estimates ranged between -6% and 16% of average accelerometer scores [58]. This notion is important because it has important implications for survey research. The concept of group-level estimates and the potential of self-report tools for surveillance research might partially explain why self-report tools are still considered to be a valuable tool in physical activity research.

### Statistical Analyses: Calibration

#### Background

Depending on the nature of physical activity questionnaires, it may be possible to generate individual calibration equations for discrete periods of time. An advantage of generating individual item regression calibration models is that context-adjusted beta weights (eg, β recess, β after school) can then be aggregated into a composite score (total weekly physical activity) by properly weighting the frequencies of each period. There are a number of other advantages of computing individual regression models for each item. In a more generic calibration approach [58], activity monitor data would not be fragmented into individual time segments. Raw item scores would be simply aggregated into a composite score and then calibrated against total MVPA measured by the accelerometer. This would cause individual items to be weighted equally in the prediction of total physical activity. The simplistic approach also inherently assumes that relationships with objective data would be similar (ie, that there would be equivalent slopes and intercepts across items). Individual equations provide a more robust calibration approach, but another key advantage is that the sample sizes can be maximized. This is directly related to the compliance criteria used to screen and process accelerometer data. A traditional calibration design would typically require participants to have worn the monitor for a complete day across an entire week whereas individual calibration would limit compliance to the availability of data for specific periods of the day. In other words, a participant that only wore the accelerometer during the evening would be excluded based on a traditional calibration approach, but with individually calibrated items his/her data would be included in the calibration of the evening item. By screening periods individually, it is possible to maximize sample size and improve statistical power. A final advantage is that it is possible to aggregate school (eg, recess, PE, commuting to school, lunch) and nonschool (eg, before school, after school, evening, Saturday, and Sunday) activity estimates to create separate composite activity scores for these time segments. This provides more utility for school leaders interested in quantifying physical activity during school. It also provides more educational value for children/parents and is more powerful for research applications.

#### YPAMS Example

Our calibration work was specifically designed to calibrate individual items from the YAP against physical activity estimates obtained from the SenseWear Armband. An example of the calibration model for activity measured by the SenseWear Armband during PE using YAP item number 2 is provided by the equation MVPA=β_0_+β_1_+β_2_+β_3_+ε_i_, in which *MVPA* is the SenseWear Armband measured percent time MVPA during PE class, *β*_*0*_ is the intercept, *β*_*1*_ is the beta weight associated with age, *β*_*2*_ is the beta weight associated with gender, *β*_*3*_ is the beta weight associated with YAP question 2 score (PE), and *ε*_*i*_ is random error (independent and normally distributed).

**Figure 5:**
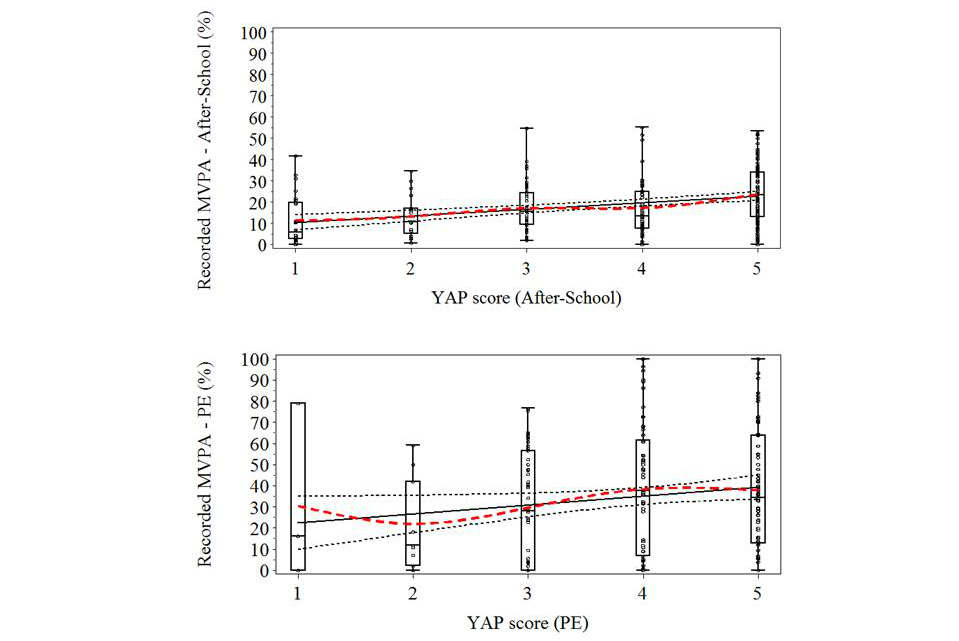
Relation between percent time in moderate-to-vigorous physical activity (MVPA) measured by the SenseWear Armband accelerometer and YAP raw scores during physical education (PE; bottom) and during after-school time (top) (n=221 participants from grades 4 to 12). The solid black line represents the line of best fit with respective 95% confidence intervals; the dashed red line fits a smooth curve across the distribution of scores. The lack of overlap between these suggests a nonlinear trend relation between percent time in MVPA and YAP scores.

This approach is particularly important because our preliminary research has demonstrated that the assumption of equivalent slopes does not hold (unpublished results). Specifically, we found that some items are related linearly to accelerometer data whereas others are not. Figure 5 provides examples of the relation between the accelerometer and 2 YAP items as a result of our preliminary work. The bottom panel shows the relation between accelerometer EE values and YAP item 2 (activity during PE) whereas the top panel shows the relation between accelerometer EE values and YAP item 6 (activity after school). Observed PE estimates of activity have a fairly linear increase from a score of 2 through a score of 4 and a plateau at the highest end of the scale, whereas the same relation for activity after school is linear across the full-item scale. These relations can be accounted for by fitting different models for each of these items and, therefore, allowing regression coefficients to vary. By calibrating individual items, we have also maximized our sample size and found that using both approaches (individual calibration vs traditional week calibration protocols) on the same data resulted in a larger sample when items were individually calibrated (n=195 vs n=148, respectively). Finally, the individual YAP items are organized in 2 activity sections to provide separate estimates of activity at school and out-of-school contexts.

### Statistical Analyses: Cross-Validation

#### Background

Rather than describing the typical validation designs used in physical activity measurement research [44,56,59-64], we provide a brief explanation on the importance of alternative statistical designs to determine the accuracy of group-level estimates obtained from self-reports. Equivalence between physical activity measures is usually determined if the average difference between the 2 instruments is not statistically significant and, therefore, not different from zero. This approach has some limitations and, importantly, tests the hypothesis that measures obtained from the 2 instruments are different. This is the reverse of what is actually being tested. The classic approach for equivalence is also too conservative because it does not specify any margin of equivalence and therefore, depending on the design of the equivalence study (eg, large sample size), it might be oversensitive to small differences between self-report and accelerometer scores [65,66].

#### YPAMS Example

In the YPAMS, we evaluate group-level agreement using bioequivalence testing procedures. This method is commonly used in medical research and is often used to determine equivalency between alternate drug substances that have been developed for the same purpose [67]. For the purpose of calibration, we tested if the difference between accelerometer and self-report estimates of MVPA were within a predefined region of equivalence, defined as 10% (other equivalence regions can be defined, such as a 20% range). The region of equivalence is rather subjective, but there are specific recommendations proposed by the Food and Drug Administration [68] that can be used for guidance. Measures can be considered “equivalent” if the 2 1-sided 95% confidence intervals for the average difference between the 2 measures are within the equivalence region (ie, 10%) of the group accelerometer estimates.

### Online Applications

#### Background

One key difference between print and online versions of a PAQ is that it is not clear if children will respond similarly to questions on a screen as they do with a print format. For example, the reliability and validity of Web-based surveys can be affected by the screen display of the survey (eg, color combinations, text background) [69,70]. The research on this topic is still limited, so it is premature to assume that youth would respond similarly to items on paper versus those on a screen. Therefore, the first step in this process is to directly evaluate possible differences between the print version and the new online version of the tool. Secondly, researchers also need to examine the feasibility of the online tool and anticipate administration, completion, and data access/sharing issues. One possible strategy to overcome unexpected challenges is to make the survey available to a subset of participants/schools to simulate the broader use of the tool.

#### YPAMS Example

The YAP was developed with online applications in mind, but separate calibration work is needed because the Web format may alter relationships and associations. We have extended our calibration methods to enhance the utility of an online version and a game version deployed within the FITNESSGRAM software. In this case, it is important to refine the calibration equations while also testing for equivalence between formats. We conducted 2 small pilot studies to test these assumptions. In Study 1, we randomized 3 grade 5 classes (n=60) to complete the 3 different versions of the YAP 2 weeks apart. We found similar test-retest coefficients for print (*r*=.86), online (*r*=.79), and game (*r*=.84), which supported the utility of the Web-based versions. In Study 2, we recruited 70 children (aged 10-15 years) to test whether the existing print-based algorithms would work with the online version of the YAP. We found that the correlations between the measured and YAP physical activity indicators were high and significant (*r*=.70, *P*<.001); however, differences between measured and estimated minutes of physical activity were larger than in our previous work. This demonstrated the feasibility and potential of the online version and suggested that the online YAP can be improved if directly calibrated. These pilot studies demonstrate the importance of successive rounds of calibration and cross-validation to refine the accuracy and utility of the instruments over time. This principle would apply to any related calibration work with other instruments.

The preceding methodology has led to substantial reductions in overall error in our calibration models. Our preliminary models demonstrated that the individual calibration method resulted in a reduction of 44% of the error we would get if items were not individually calibrated. Although the results demonstrate good utility, our approach is to sequentially refine and improve the precision over time by increasing the size and diversity of the sample while also taking into account other factors, such as seasonality, urbanicity, and regional differences. These issues are introduced in the next section.

## Additional Considerations

The inherent goal with any measurement instrument is to capture variation between individuals while minimizing sources of systematic and/or random error. Systematic error (bias) occurs consistently over measurements and may depend on subject-level attributes, whereas random error is the inconsistent variability among individuals or measurements. Unlike many other more stable health indicators, physical activity is a behavior that varies naturally from day to day and from season to season for an individual [71,72] and this makes it particularly challenging to assess. Assessing physical activity and sedentary behavior in youth is even more challenging due to maturation differences and sporadic activity patterns [73].

Considerable advances have been made in the use of objective tools (eg, accelerometers) in the past decade and there are similar opportunities to advance the quality and utility of self-report measures. The observed “error” in self-report instruments is often attributed to participant’s bias or inability to recall information. These are certainly important factors, but it is also important to acknowledge that error is contributed directly by poorly designed questions, weak scoring methods, and an inability to accurately characterize and quantify the information that is provided. With calibration methods, it is possible to convert self-reported data into estimates that more closely model estimates that are obtained from objective sources. The information in this paper describes lessons learned through our exploration of ways to improve physical activity assessment protocols [44,73,74] and our interest in systematic efforts to improve the utility of self-report measures [13,22,58,75,76].

Another issue that needs to be tested is whether calibration would vary by region/season. Activity profiles vary by season and youth may respond differently about physical activity patterns in each season. Our design incorporates the natural variability across seasons and we are working to evaluate possible differences across regions. By replicating established calibration measurement protocols on independent samples of youth from different communities (at the same time), it will be possible to eventually determine whether calibration models hold when used in different regions. Cross-validation of the calibration equations originally developed would provide information about how the models hold in more diverse samples with different weather and culture. If there are differences, we anticipate being able to eventually develop more robust measurement models similar to those used in our adult calibration work [77].

A final need for continued development is to test the utility of the tool under less-controlled conditions. The original data for our calibration models were also collected by trained staff that could prompt youth to pay attention to aspects of the instrument. This helps to improve internal validity of the study, but may detract from external validity. It will eventually be important to test the accuracy of this tool under more real-world conditions. The distinction is similar to the way that preliminary calibration studies with accelerometers used controlled laboratory conditions and tested only locomotor activities. Subsequent studies conducted under free-living conditions demonstrated that it is considerably harder to accurately capture the diverse range of activities that children perform. Similarly, it will be more difficult to obtain accurate self-report data when used in less-controlled school settings. These points should not be interpreted as unsolvable problems, but as challenges to be overcome. We expect that calibration methods will enable accurate group-level estimates of physical activity to provide more accurate reports of age and gender patterns of physical activity; however, we acknowledge that it will remain difficult to obtain accurate individual estimates. Again, this is the same challenge faced by researchers working to refine the accuracy of objective measures. Calibration equations for monitors may have utility for group estimation, but accurately estimating individual data remains more elusive [56,57].

The refinement of prediction algorithms for the YAP has important school health and public health implications. Evaluating and refining the calibrations of the online and game-based versions of the YAP will facilitate planned adoption within the FITNESSGRAM program. The newly established Presidential Youth Fitness Program (PYFP) recently established FITNESSGRAM as the national fitness test so this calibrated physical activity assessment tool would potentially provide a way to capture physical activity levels in schools throughout the country. Because calibration equations can be incorporated directly into the online tool, it would be possible to provide immediate feedback to youth and to generate automated school-level reports with aggregated data. Fully refined online versions of the YAP would enable broader use by schools interested in tracking participation in MVPA as part of district or state programming. The tools would also facilitate research applications on school activity (for epidemiology studies or behavioral interventions).

We anticipate opportunities to also explore potential for use in cross-cultural studies and international comparisons [78]. At this level, there will be language barriers and, expectedly, more cross-cultural differences that will need to be addressed. There are several procedures involved in this process and they target language barriers initially by using well-known translation procedures [79]. The International Physical Activity Questionnaire is a good example of the result of a combined effort to standardize and promote physical activity across the globe [80]. Several studies have used this tool and some examples of cross-validation studies include study populations of older adults in Japan [81], adults in Greenland [82], and adolescents in Vietnam [83]. We envision that similar developments and refinement are possible with the YAP.

Ultimately, we view this calibration work as a long-term, iterative process that will lead to continued and incremental improvements over time. The principles described in this paper utilize recommended practices to reduce measurement error with self-report measures [45] as well as recommended steps to test the validity of self-report instruments [46]. They provide a good guide to ensure that the work progresses in a systematic way. Although the calibration principles described here are specific for the YAP, the concepts and methods may have utility for researchers interested in similar calibration work with other tools or Web-based applications. There is an increased interest in Web applications of physical activity surveys [14-17,19,84]; however, to our knowledge no research has described a systematic process to develop, calibrate, and disseminate the use of such assessments.

## Multimedia Appendix 1

Youth Activity Profile Questionnaire (paper-version).

## Abbreviations

EE: energy expenditure
MET: metabolic equivalent of task
MVPA: moderate-to-vigorous physical activity
PAQ: Physical Activity Questionnaire
PE: physical education
PYFP: Presidential Youth Fitness Program
YAP: Youth Activity Profile
YPAMS: Youth Physical Activity Measurement Study
