# Supplementary material for: Web-Based Assessments of Physical Activity in Youth: Considerations for Design and Scale Calibration
Source: J Med Internet Res. 2014 Dec 1;16(12):e269. doi: 10.2196/jmir.3626 (PMC4275492; doi:10.2196/jmir.3626)
Supplement: Supplementary file 1 [file jmir_v16i12e269_app1.pdf]

Youth Activity Profile

Before you begin, it is important to get some basic information about your school and about you.

Circle your Gender: Male Female

Circle your School Level: Elementary School Middle School High School

Circle your Grade: 3 4 5 6 7 8 9 10 11 12

ID _____________

How many days each week do you have PE?
a. 0 days (never)
b. 1 day
c. 2 days
d. 3 days
e. 4 days
f. 5 days

How many break/study hall periods do you have per day?
a. 0
b. 1
c. 2
d. 3
e. 4

How many times last week did you attend sessions or practices for sports or structured physical activities that were led by a coach, instructor or leader
a. 0
b. 1
c. 2
d. 3
e. 4
f. 5 or more

The Youth Activity Profile will ask you about the time you spend being active (both in school and out of school) and the time you spend being sedentary.

- **Physical activities** are things that involve a lot of walking, running or moving around. It includes biking and dancing as well as sports or outdoor play that involves a lot of moving around.
- **Sedentary activities** are things such as watching TV, or playing video games, computer games, or hand-held games that you do in your free time. It does NOT include the time you spend sitting while eating or while doing homework.

Most questions will ask you only to think about the <u>last 7 days</u> but a few questions will ask about what you typically do (on a normal week). <u>There are no right or wrong answers so provide honest answers.</u>

Youth Activity Profile
Activity Levels - at School

Activity Levels - at School. These questions ask about your physical activity at school. This includes physical education but you may also be active on your way to school, during breaks, or at lunch. Answer the questions based on your physical activity at school in the <u>last 7 days</u>.

1. Activity To School: How many days did you **walk or bike to school**? *(If you can't remember, try to estimate)*
a. 0 days (never)
b. 1 day
c. 2 days
d. 3 days
e. 4-5 days (most every day)

2. Activity during Physical Education Class: During **physical education**, how often were you running and moving as part of the planned games or activities? *(If you didn't have PE, choose "I didn't have physical education")*
a. I didn't have physical education
b. Almost none of the time
c. A little bit of the time
d. A moderate amount of time
e. A lot of the time
f. Almost all of the time

3. Activity during Breaks/Study Hall: During **breaks/study hall**, how often were you playing sports, walking, running, or playing active games? *(If you didn't have a break at school, choose "I didn't have breaks/study hall")*
a. I didn't have breaks/study hall
b. Almost none of the time
c. A little bit of the time
d. A moderate amount of time
e. A lot of the time
f. Almost all of the time

<u>Elementary Version:</u> **Activity during Recess:** During **recess**, how often were you playing sports, walking, running, or playing active games? *(If you didn't have a recess at school, choose "I didn't have recess").*

Changed option a: I didn't have recess

4. Activity during Lunch: During **lunch break**, how often were you moving around, walking or playing? *(If you didn't have a lunch break at school, choose "I didn't have lunch breaks")*
a. I didn't have lunch breaks
b. Almost none of the time
c. A little bit of the time
d. A moderate amount of time
e. A lot of the time
f. Almost all of the time

5. Activity from School: How many days often did you **walk or bike from school**? *(If you can't remember, try to estimate)*
a. 0 days (never)
b. 1 day
c. 2 days
d. 3 days
e. 4-5 days (most every day)

Youth Activity Profile
Activity Levels at Home

Activity Levels - Outside of School. These questions ask about your overall levels of physical activity during different periods of time (outside of school time). This would include structured exercise or sport activities as well as activity playing with friends, dancing or doing work/chores. Answer the questions based on your physical activity outside of school <u>in the last 7 days</u>.

6. Activity before School: How many days **before school (6:00-8:00 am)** did you do some form of physical activity for at least 10 minutes? (This includes activity at home NOT walking or biking to school)
a. 0 days
b. 1 day
c. 2 days
d. 3 days
e. 4 to 5 days

7. Activity after School: How many days **after school (between 3:00 -6:00 pm)** did you do some form of physical activity for at least 10 minutes? (This can include playing with your friends/family, team practices or classes involving physical activity but *NOT walking or biking home from school*)
a. 0 days
b. 1 day
c. 2 days
d. 3 days
e. 4 to 5 days

8. Activity on Weeknights: How many **school evenings (6:00-10:00 pm)** did you do some form of physical activity for at least 10 minutes? (This can include playing with your friends/family, team practices or classes involving physical activity but *NOT walking or biking home from school*)
a. 0 days
b. 1 day
c. 2 days
d. 3 days
e. 4 to 5 days

9. Activity on Saturday: How much physical activity did you do last **Saturday**? (*This could be for exercise, work/chores, family outings, sports, dance, or play. If you don't remember, try to estimate)*
a. No activity (0 minutes)
b. Small amount of activity (1 to 30 minutes)
c. Small to Moderate amount activity (31 to 60 minutes)
d. Moderate to Large amount of activity (1 to 2 hours)
e. Large amount of activity (more than 2 hours)

10. Activity on Sunday: How much physical activity did you do last **Sunday**? (*This could be for exercise, work/chores, family outings, sports, dance, or play. If you don't remember, try to estimate)*
a. No activity (0 minutes)
b. Small amount of activity (1 to 30 minutes)
c. Small to Moderate amount activity (31 to 60 minutes)
d. Moderate to Large amount of activity (1 to 2 hours)
e. Large amount of activity (more than 2 hours)

Youth Activity Profile

Sedentary Habits

These questions ask about time spent resting and sitting. You probably sit while eating, doing homework, or playing musical instruments. But you also may spend time sitting while watching TV, playing video games, using the computer or using your phone, or iTouch/iPad). Answer these questions about the time you spent sitting during these activities <u>in the past 7 days</u>.

11. TV Time: How much time did you spend **watching TV** outside of school time (*This includes time spent watching movies or sports but NOT time spent playing video games*).

a. I didn't watch TV at all
b. I watched less than 1 hour per day
c. I watched 1 to 2 hours per day
d. I watched 2 to 3 hours per day
e. I watched more than 3 hours per day

12. Video Game Time: How much time did you spend **playing video games** outside of school time? (*This includes games on Nintendo DS, wii, Xbox, PlayStation, iTouch, iPad, or games on your phone*)

a. I didn't really play at all
b. I played less than 1 hour per day
c. I played 1 to 2 hours per day
d. I played 2 to3 hours per day
e. I played more than 3 hours per day

13. Computer Time: How much time did you spend using **computers** outside of school time? (*This <u>doesn't</u> include home work time but includes time on Facebook as well as time spent surfing the internet, instant messaging, playing online video games or computer games*)

a. I didn't really use the computer at all
b. I used a computer less than 1 hour per day
c. I used a computer 1 to 2 hours per day
d. I used a computer 2 to3 hours per day
e. I used a computer more than 3 hours per day

14. Phone / Text Time: How much time did you spend using your **cell phone** after school? (*This includes time spent talking or texting*).

a. I didn't really use a cell phone
b. I used a phone less than 1 hour per day
c. I used a phone 1 to 2 hours per day
d. I used a phone 2 to 3 hours per day
e. I used a phone more than 3 hours per day

15. Overall Sedentary Habits: Which of the following best describes your **<u>typical</u>** sedentary habits at home? *(Try to think about a typical week and not just last week)*

a. I spent almost none of my free time sitting
b. I spent little time sitting during my free time
c. I spent a moderate amount of time sitting during my free time
d. I spent a lot of time sitting during my free time
e. I spent almost all of my free time sitting
